# Transcranial Evoked Potentials Can Be Reliably Recorded with Active Electrodes

**DOI:** 10.3390/brainsci11020145

**Published:** 2021-01-22

**Authors:** Marco Mancuso, Valerio Sveva, Alessandro Cruciani, Katlyn Brown, Jaime Ibáñez, Vishal Rawji, Elias Casula, Isabella Premoli, Sasha D’Ambrosio, John Rothwell, Lorenzo Rocchi

**Affiliations:** 1 Department of Human Neurosciences, University of Rome “Sapienza”, 00185 Rome, Italy; mancu94@gmail.com (M.M.); valerio.sveva@gmail.com (V.S.); 2 Neurology, Neurophysiology and Neurobiology Unit, Department of Medicine, Università Campus Bio-Medico di Roma, 00128 Rome, Italy; alessandrocruciani93@hotmail.it; 3 Department of Clinical and Movement Neurosciences, UCL Queen Square Institute of Neurology, University College London, London WC1N 3BG, UK; kate.brown@uwaterloo.ca (K.B.); vishal.rawji.11@alumni.ucl.ac.uk (V.R.); j.rothwell@ucl.ac.uk (J.R.); 4 Department of Bioengineering, Faculty of Engineering, Imperial College London, London SW7 2AZ, UK; j.ibanez-pereda@imperial.ac.uk; 5 Non-Invasive Brain Stimulation Unit, IRCCS Santa Lucia Foundation, 00142 Rome, Italy; elias.casula@gmail.com; 6 Institute of Psychiatry, Psychology, and Neuroscience, King’s College London, London SE5 8AF, UK; isabella.premoli@kcl.ac.uk; 7 Chalfont Centre for Epilepsy, Chalfont St. Peter SL9 0RJ, UK; s.dambrosio@ucl.ac.uk; 8 Department of Clinical and Experimental Epilepsy, UCL Queen Square Institute of Neurology, University College London, London WC1N 3BG, UK

**Keywords:** transcranial magnetic stimulation, electroencephalography, TMS-EEG, transcranial evoked potentials, motor evoked potentials, active electrodes, neurophysiology, EEG artefacts, independent component analysis

## Abstract

Electroencephalographic (EEG) signals evoked by transcranial magnetic stimulation (TMS) are usually recorded with passive electrodes (PE). Active electrode (AE) systems have recently become widely available; compared to PE, they allow for easier electrode preparation and a higher-quality signal, due to the preamplification at the electrode stage, which reduces electrical line noise. The performance between the AE and PE can differ, especially with fast EEG voltage changes, which can easily occur with TMS-EEG; however, a systematic comparison in the TMS-EEG setting has not been made. Therefore, we recorded TMS-evoked EEG potentials (TEPs) in a group of healthy subjects in two sessions, one using PE and the other using AE. We stimulated the left primary motor cortex and right medial prefrontal cortex and used two different approaches to remove early TMS artefacts, Independent Component Analysis and Signal Space Projection—Source Informed Recovery. We assessed statistical differences in amplitude and topography of TEPs, and their similarity, by means of the concordance correlation coefficient (CCC). We also tested the capability of each system to approximate the final TEP waveform with a reduced number of trials. The results showed that TEPs recorded with AE and PE do not differ in amplitude and topography, and only few electrodes showed a lower-than-expected CCC between the two methods of amplification. We conclude that AE are a viable solution for TMS-EEG recording.

## 1. Introduction

The concurrent use of transcranial magnetic stimulation and electroencephalography (TMS-EEG) has recently emerged as a powerful and widely used non-invasive stimulation method to investigate human brain function [1,2,3]. Modern amplifiers have been designed to reduce the TMS artefact in the electroencephalographic (EEG) signal, allowing for the characterization of cortical responses, starting only few milliseconds after the TMS stimulus [2,4,5]. Signal recording in TMS-EEG usually employs passive recording electrodes (PE); active electrodes (AE) are a more recent introduction in electrophysiology. Whereas PE act as simple recording sites, without changes in the physical signal, AE entail preamplification directly at the electrode stage. This has some practical advantages, such as the potential to reduce electrical line noise and provide better data at higher interelectrode impedance [6]. Other benefits of the use of AE, compared to PE, include a lower preparation time and ease of montage, which results in decreased discomfort for subjects, this feature being particularly useful in studies on clinical populations. Despite these advantages, AE have not yet been used in TMS-EEG studies, with few exceptions [7]. While it would be expected that the two technologies yield similar data quality, slight differences emerged between PE and AE in a study investigating event-related potentials (ERP) with a P3 oddball paradigm [8]. Notably, the mentioned capability of AE to provide clean signals with suboptimal impedance levels seems to be limited when the EEG exhibit sudden voltage changes, such as with ERPs; this may be due to a slower slew rate (i.e., the capability of an amplifier to quickly and accurately follow changes in voltage) at the preamplification stage. This issue might be relevant when recording TMS-EEG signals, due to the rapidly changing temporal profile of TMS-induced EEG responses and artefacts [9,10]. Overall, these findings warrant a formal comparison between PE and AE in the TMS-EEG setting.

In the present study, we aimed to compare the performance of AE and PE systems in recording TMS-induced EEG signals after stimulation of the primary motor cortex (M1) and medial prefrontal cortex (mPFC), which are commonly used stimulation sites in TMS-EEG studies. To do so, we calculated significant differences and similarities at the map level between the transcranial evoked potential (TEP) recorded with AE and PE in a sample of healthy subjects. We also ran an efficiency analysis, in which we estimated the ability of each system to approximate the final TEP with a smaller number of trials, similar to previous work [8]. As a consensus on the optimal pre-processing is lacking [1], the EEG analyses were performed with two different pipelines, each one entailing a different method to remove early, TMS-locked artefacts: Independent Component Analysis (ICA) and Signal Space Projection—Source Informed Recovery (SSP-SIR).

## 2. Materials and Methods

### 2.1. Participants

Eight right-handed healthy subjects [11] (age 31.63 ± 3.96, five male, three female) participated in this study. The subjects had no history of neurological or psychiatric diseases and were not taking drugs active at the central nervous system. All the procedures were performed in accordance with the Declaration of Helsinki and approved by the human subjects review board of the University College London (protocol code 10037/001). The participants gave written informed consent prior to the experimental session.

### 2.2. Transcranial Magnetic Stimulation and Electromyography

**Figure 1:**
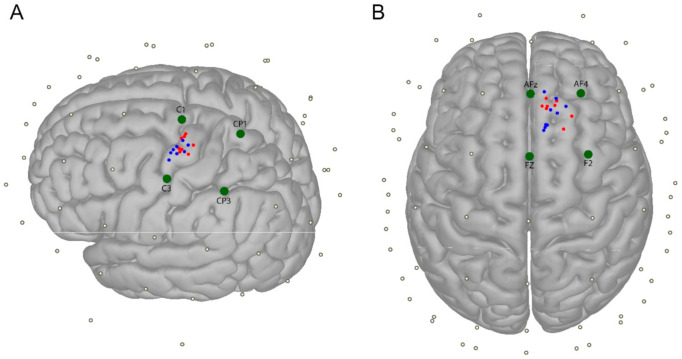
Distribution of stimulation points in M1 (**A**) and medial prefrontal cortex (mPFC) (**B**), in the two experimental sessions (PE: blue dots; AE: red dots), represented in the MNI space by using a template cortical surface. Green dots represent the four electrodes surrounding the stimulated area, which were used for the efficiency analysis (see text for details).

For TMS, a monophasic stimulus was delivered through a 70-mm figure-of-eight coil connected to a Magpro X100 magnetic stimulator (MagVenture, Farum, Denmark), with the handle backwards at 45° to the midline, inducing current in the posterior-anterior direction [12]. To obtain the M1 “hot spot”, the coil was systematically changed in position until the largest motor evoked potential (MEP) was obtained in the contralateral first dorsal interosseous (FDI) muscle, as commonly done for TMS investigations involving M1 [13,14] (Figure 1). For the mPFC, the stimulation point approximately corresponded to the right superior frontal gyrus (Figure 1), with the coil handle pointing posteriorly.

The stimulation locations (left M1, right mPFC) were sampled in MNI space and the coil was maintained in the correct position throughout the stimulation by using a Brainsight neuro-navigation system (Rogue Research Inc., Montreal, QC, Canada) coupled with a Polaris Spectra optical measurement system (Northern Digital Inc., Waterloo, ON, Canada). An estimated MRI scan in the MNI space was used for all the participants [15]. MNI coordinates during stimulation were sampled in each TMS trial in order to calculate the error in coil position (linear, angular, and twist errors). Stimulation intensity for left M1 was 90% of the resting motor threshold (RMT) measured on the left hemisphere, whereas TMS of the right mPFC was delivered at an intensity equal to 120% RMT of the right hemisphere, assuming a higher excitability of the former [16]. The RMT was defined as the intensity necessary to obtain motor evoked potentials (MEP) of at least 50 μV amplitude in roughly five out of 10 trials [12,17]. Electromyographic (EMG) signals from the FDIs were recorded by means of 10 mm diameter Ag/AgCl cup electrodes, were sampled at 5 kHz with a CED 1401 A/D laboratory interface (Cambridge Electronic Design, Cambridge, UK), amplified (gain 1000×) and filtered (bandwidth 5 Hz − 2 kHz) with a Digitimer D360 (Digitimer Ltd., Welwyn Garden City, Hertfordshire, UK).

### 2.3. Electroencephalographic Recording and Analysis

EEG was recorded using a DC-coupled TMS compatible amplifier (Brainamp DC, Brain Products GmbH, Gilching, Germany). The scalp of each subject was prepared for EEG recording using an abrasive/conductive gel (V17 Abralyt 2000). The EEG signal was digitized with a sampling frequency of 5 kHz from the following electrodes: FP1, FPz, FP2, AF7, AF3, AF4, AF8, F7, F5, F3, F1, Fz, F2, F4, F6, F8, FT7, FC5, FC3, FC1, FC2, FC4, FC6, FT8, T7, C5, C3, C1, Cz, C2, C4, C6, T8, TP9, TP7, CP5, CP3, CP1, CPz, CP2, CP4, CP6, TP8, TP10, P7, P5, P3, P1, Pz, P2, P4, P6, P8, PO7, PO3, POz, PO4, PO8, O1, Oz, O2, FCz, AFz. Depending on the experimental session (see below), we used either passive (EasyCap) or low-profile active (ActiCap) electrodes from the same manufacturer (Brain Products GmbH, Gilching, Germany). Two further electrodes were placed on the forehead as online reference and ground. Impedances were kept lower than 5 kΩ during the whole experiment.

Offline EEG pre-processing was performed with EEGLAB 14.1.1 [18], with the addition of some functions included in the TMS-EEG signal analyzer (TESA) toolbox [19], all running in MATLAB environment (Version 2018b, MathWorks Inc., Natick, USA). EEG signal recorded during TMS was epoched (−1.3 to 1.3 s) using a baseline from −1000 to −10 ms and the TMS artefact was removed from −5 to 12 ms around the TMS pulse. The epochs were visually inspected and those with excessively noisy EEG were excluded. After epoching, the early, TMS-locked artefacts [20] were removed by means of a fastICA algorithm; only the 15 components explaining the largest variance were plotted, in a time window ranging from −200 to 500 ms, and only those reflecting residual scalp muscle or decay were eliminated after visual inspection, based on time, frequency, scalp distribution, and amplitude criteria [21,22]. Subsequently, the signals were down-sampled to 1000 Hz, band-pass (1–100 Hz) and band-stop (48–52 Hz) filtered with a fourth order Butterworth filter. The epochs were restricted to (−1 to 1 s) in order to reduce possible edge artefacts, and a round of SOUND (source-estimate-utilizing noise-discarding algorithm) [23] was applied to further clean the signal (λ = 0.1, 20 iterations), as previously done with TMS-EEG data [24]. A second round of fastICA was then performed, plotting the full epoch length, to remove residual artefacts non time-locked with the TMS pulse (e.g., spontaneous eyeblinks and continuous muscle activity). Lastly, the EEG signals were re-referenced to the common average reference. This pre-processing pipeline will be referred to as ICA-SOUND-ICA (ISICA).

As a consensus regarding the best pre-processing method for TMS-EEG is lacking, we also used an alternative solution to remove the early, TMS-locked artefacts, i.e., SSP-SIR [25], which replaced the first round of ICA. We deemed this approach important since early artefacts, in particular those caused by the direct activation of scalp muscles, might be different between the two stimulated areas, for anatomical reasons, and between the two recording sessions, since TMS performed with AE requires stronger TMS pulses, due to the added thickness of recording electrodes (see below). The second pipeline will be referred to as SSP-SIR-SOUND-ICA (SSICA).

### 2.4. Experimental Design

The participants were seated in a comfortable chair, in a quiet room, with forearms resting on a pillow that was placed on their lap. They were asked to fixate a white cross (6 × 6 cm) in the middle of a blank screen placed in front of them, to avoid eye movements during the EEG recordings. The subjects wore earphones that continuously played a masking noise, which was composed of white noise mixed with specific time-varying frequencies of the TMS click, to minimize the auditory evoked potential (AEP) caused by the latter [26]. The intensity of the masking noise was adjusted for each participant by increasing the volume until the subject was sure that s/he could no longer hear the TMS click, or as much as tolerated (always below 90 dB) [27]. In order to further reduce the impact of the TMS click on EEG signals, we placed ear defenders (SNR = 30) on top of the earphones; this approach has been proven to be effective in suppressing the AEP that is caused by TMS [7]. Additionally, a 0.5 cm foam layer was placed underneath the coil to minimize bone conduction of the TMS click and the scalp sensation that is caused by coil vibration [7,28].

The participants underwent two recording sessions in randomized order, on two separate days, at least one week apart, where EEG was recorded with either AE or PE. In each session, two blocks of 150 TMS stimuli were delivered, one on the left M1 and the other on the right mPFC, as described above. At the end of each session, the subjects were asked to score the loudness of the TMS click for each recording block by means of a visual analogue scale (VAS), ranging from 0 (no perception) to 10 (maximal perception). The same scoring was used for discomfort (0 = no discomfort; 10 = maximal discomfort).

### 2.5. Data Analysis and Statistics

The following variables were compared, for each stimulated area, in AE vs. PE recording conditions: RMT, stimulation intensity, VAS scores for residual auditory sensation and discomfort, MNI coordinates (x, y, z), error in coil positioning (linear, angular, twist). A comparison was also made between the RMT measured on the right and left hemisphere within each experimental session. Because most of the variables were non-normally distributed (*p* values of the Shapiro-Wilk test < 0.05) and due to the small sample size, the mentioned comparisons were performed by means of the Wilcoxon Signed Rank test, while using SPSS version 25 (SPSS Inc., Chicago, IL, USA).

The EEG signals were analyzed using custom scripts written in Matlab, version 2018b (MathWorks Inc., Natick, MA, USA). The general aim of the following analyses was to investigate differences and similarities of TEPs obtained with PE and AE, separately considering the two stimulated areas (M1, mPFC) and the two pre-processing pipelines used to remove early, TMS-locked artefacts (ISICA, SSICA). In the first analysis, possible differences in signals amplitude were assessed. To do this, the TEPs were first divided into discrete time windows, identified based on the grand average between the signals recorded at the same stimulation site in the two sessions [7]. The average TEP amplitude was calculated in each time window and values from each electrode were compared between recording sessions by means of paired t-tests, with false discovery rate (FDR) correction for multiple comparisons.

In a second analysis, we sought to investigate similarities between the signals recorded with PE and AE. To do so, we calculated the concordance correlation coefficient (CCC) between TEPs in each time window (obtained as previously explained). The CCC is a form of intraclass correlation coefficient that takes both the covariance and the absolute distance between two distributions into account. It is calculated as follows:

$$CCC=\frac{2\sigma_{12}}{\sigma_{1}^{2}+\;\sigma_{2}^{2}+{\left({µ}_{1}-\;{µ}_{2}\right)}^{2}\;}$$

where $\sigma_{12}$ is the covariance between two distributions, $\sigma_{x}^{2}$ is the variance of distribution *x*, and ${µ}_{x}$ is the average of distribution *x* [29]; this method has been previously used in the TMS-EEG setting [7,30]. The statistical analysis entailed an extreme-corrected permutation approach, where the CCC was first calculated between conditions in each electrode and time window. After this, a custom distribution of CCC values was built by shuffling each subject’s trials between the two conditions (PE, AE) 1000 times. The CCC values found in our sample were deemed significant if their values fell in the lower fifth percentile of the custom distribution, divided by the number of time windows, to account for type I error across multiple observations. We then applied extreme correction to solve the problem of multiple comparisons across electrodes. To this aim, we built a distribution using the lowest CCC values found among the 63 electrodes in each permutation: only the CCC values that fell in the lowest fifth percentile of this distribution, divided again by the number of time windows, were considered statistically significant [31]. This resulted in a one-tailed statistics, aimed at identifying those groups of electrodes which would show a CCC value smaller than the expected under the null-hypothesis (i.e., assuming that the two sessions were performed with the same electrode type). Lastly, we performed an efficiency analysis that aimed to clarify possible differences between PE and AE in the number of trials needed to approximate the signal obtained by averaging all trials, as done previously [8]. Our signal of interest was the TEP calculated by averaging signals from the four electrodes surrounding the stimulation site [32,33,34] (Figure 1); this process involved different electrodes, based on where TMS was applied (M1: C1, C3, CP1, CP3; mPFC: Fz, F2, AFz, AF4). For each session (PE, AE) and pre-processing pipeline (ISICA, SSICA), we calculated the CCC between the TEP derived from n number of trials and the TEP derived from all the trials, with n going from 1 to the number of trials of the subject with the lowest number of residual epochs in each session (125 for M1, 135 for mPFC); this limitation is intrinsic to the type of statistic used, since each comparison was performed in a within-subject fashion. In order to smooth the results, we repeated the process 100 times, each one switching the individual subject’s trial order, and then averaged the 100 curves built in this way. The difference between AE and PE curves was then considered as our variable of interest. This was compared to the distribution of differences derived from repeating the process 1000 times, each time switching a random number of trials between AE and PE conditions, within each subject. Differences in CCC observed between the PE and AE curves were considered to be significant if they fell in the 5th highest percentile of the distribution. In order to correct for multiple comparisons, we adopted a cluster-based permutation approach [35]. The largest clusters in adjacent time points in each permutation were sorted in ascending order; we calculated the 95th percentile of this cluster size distribution and used it as a threshold for significance.

## 3. Results

The test sessions were tolerated by all subjects and no adverse effects were reported. RMT in the left and right hemispheres did not show significant differences in both the PE (left vs. right: 45.75 ± 10.38, 45.5 ± 9.11; Z = 0.021, *p* = 0.932) and AE (51.63 ± 8.30 vs. 52.125 ± 10.04, Z = −0.315, *p* = 0.752) sessions. Table 1 reports the results of the Wilcoxon Signed Rank tests, as well as descriptive statistics of the variables compared between the two sessions.

**Figure 2:**
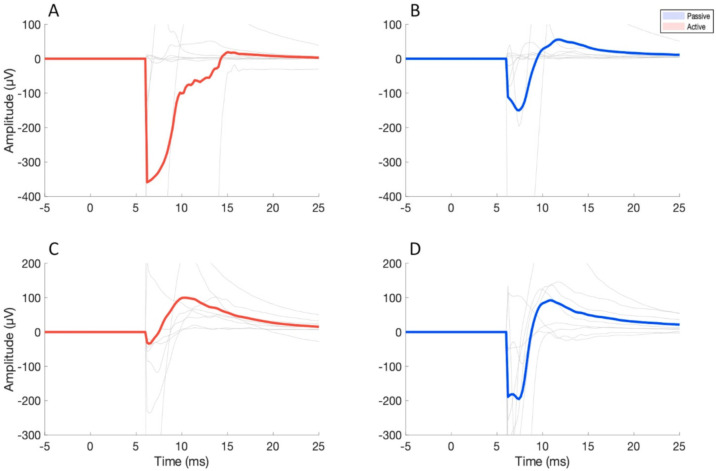
Illustration of grand-averaged electroencephalographic (EEG) artefact caused by direct scalp muscle activation by TMS on M1 (**A**,**B**) and mPFC (**C**,**D**). Red lines represent AE recordings, while blue lines refer to PE recordings. Light grey lines indicate are individual subjects’ traces. Signals were cut before 7 ms to eliminate the artefact due the TMS pulse itself and residual current decay. M1 artefact was recorded from FT7, while for mPFC FP2 electrode was chosen, according to the topography of the maximal amplitude of the EMG.

The TMS intensity was significantly higher in the AE session compared to the PE one, for both stimulated areas, probably because the low-profile AE used in the present study are thicker than the PE, thus effectively increasing the distance between coil and cortex. The higher stimulation intensity led to slightly higher VAS scores for auditory perception when AE were used; however, the comparison with PE did not reach statistical significance, pointing to an effective masking of the TMS click. Interestingly, the higher TMS intensity that was used when AE were employed did not translate into higher VAS scores for discomfort, probably because of the larger coil to scalp distance when using AE. The complex relationship between TMS intensity and coil to scalp distance is reflected in the artefact caused by direct activation of scalp muscle, which was slightly larger with PE, compared to AE, for M1 stimulation, but opposite for mPFC stimulation (Figure 2).

The two sessions did not significantly differ in terms of location of stimulated areas (Figure 1) and precision in coil positioning, as demonstrated by statistics on MNI coordinates and coil position error (Table 1).

**Figure 3:**
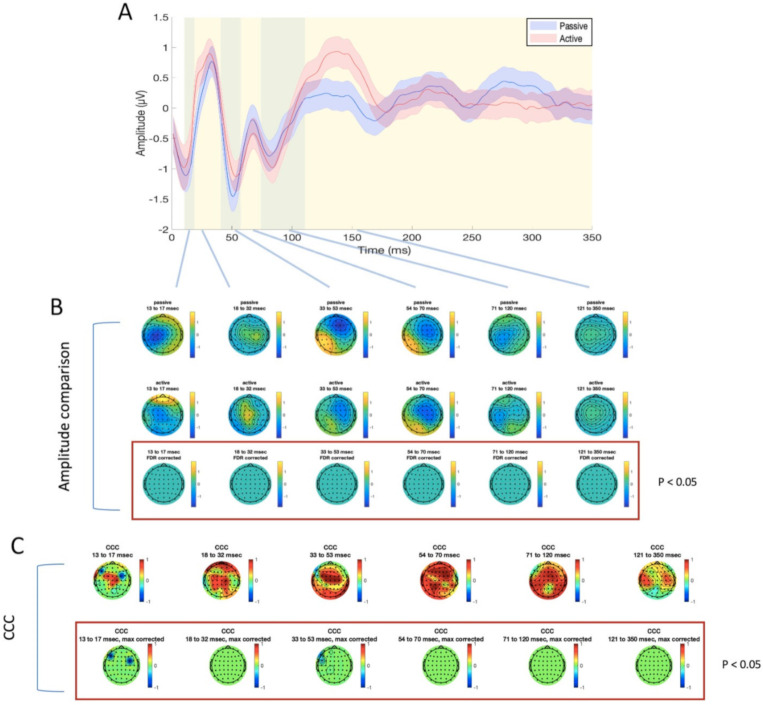
Differences and similarities between PE and AE sessions in the M1 ISICA condition. (**A**) transcranial evoked potential (TEP) averaged in the four electrodes surrounding the stimulation site (C1, C3, CP1, CP3), for passive (blue line) and active (red line) electrodes. Shaded areas indicate the standard error of the mean; green and yellow panels refer to the time windows of interest which were used for amplitude comparison and concordance correlation coefficient (CCC). (**B**) amplitude comparison between the signals obtained with PE (upper row) and AE (middle row). No statistically significant differences were found (lower row). (**C**) CCC analysis (raw CCC values are illustrated in the upper row, while statistically corrected values are visualized in the lower row). Only a few electrodes showed significantly lower CCC than expected.

**Figure 4:**
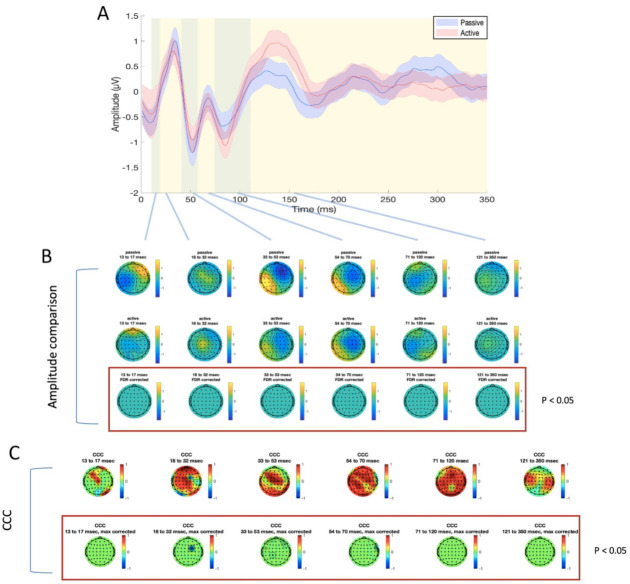
Differences and similarities between PE and AE sessions in the M1 SSICA condition. (**A**) TEP averaged in the four electrodes surrounding the stimulation site (C1, C3, CP1, CP3), for passive (blue line) and active (red line) electrodes. Shaded areas indicate the standard error of the mean; green and yellow panels refer to the time windows of interest which were used for amplitude comparison and CCC. (**B**) amplitude comparison between signals obtained with PE (upper row) and AE (middle row). No statistically significant differences were found (lower row). (**C**) CCC analysis (raw CCC values are illustrated in the upper row, while statistically corrected values are visualized in the lower row). Only a few electrodes showed significantly lower CCC than expected.

**Figure 5:**
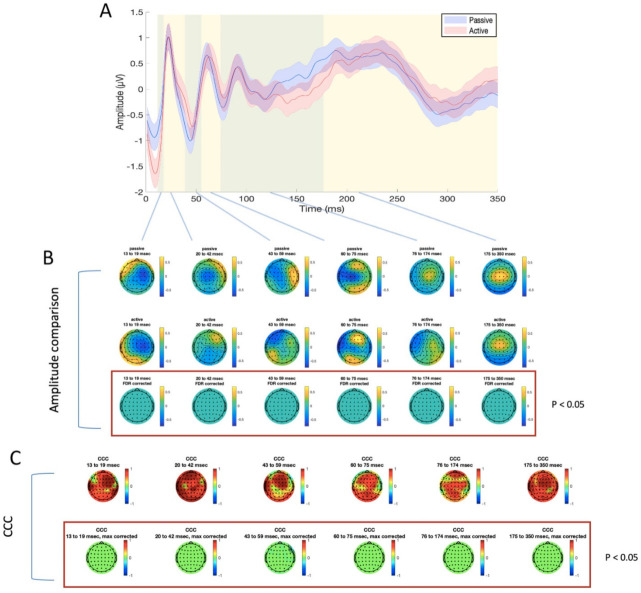
Differences and similarities between PE and AE sessions in the mPFC ISICA condition. (**A**) TEP averaged in the four electrodes surrounding the stimulation site (FZ, AF2, AFZ, AF4), for passive (blue line) and active (red line) electrodes. Shaded areas indicate the standard error of the mean; green and yellow panels refer to the time windows of interest which were used for amplitude comparison and CCC. (**B**) amplitude comparison between signals obtained with PE (upper row) and AE (middle row). No statistically significant differences were found (lower row). (**C**) CCC analysis (raw CCC values are illustrated in the upper row, while statistically corrected values are visualized in the lower row). Only one electrode showed significantly lower CCC than expected.

**Figure 6:**
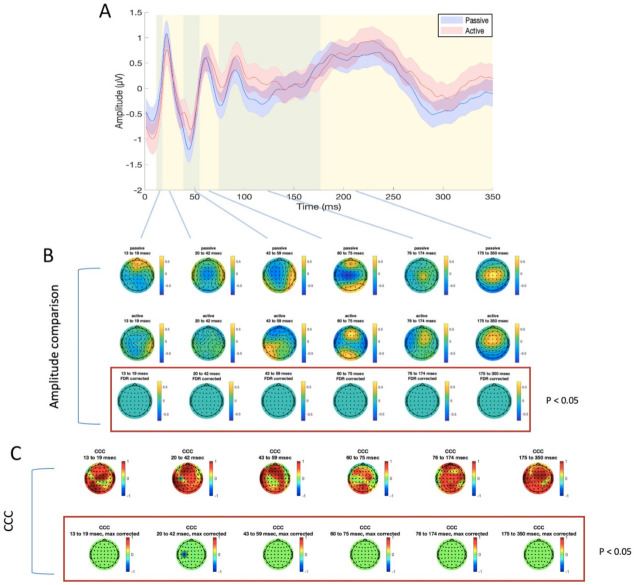
Differences and similarities between PE and AE sessions in the mPFC SSICA condition. (**A**) TEP averaged in the four electrodes surrounding the stimulation site (FZ, AF2, AFZ, AF4), for passive (blue line) and active (red line) electrodes. Shaded areas indicate the standard error of the mean; green and yellow panels refer to the time windows of interest which were used for amplitude comparison and CCC. (**B**) amplitude comparison between signals obtained with PE (upper row) and AE (middle row). No statistically significant differences were found (lower row). (**C**) CCC analysis (raw CCC values are illustrated in the upper row, while statistically corrected values are visualized in the lower row). Only one electrode showed a significantly lower CCC than expected.

**Figure 7:**
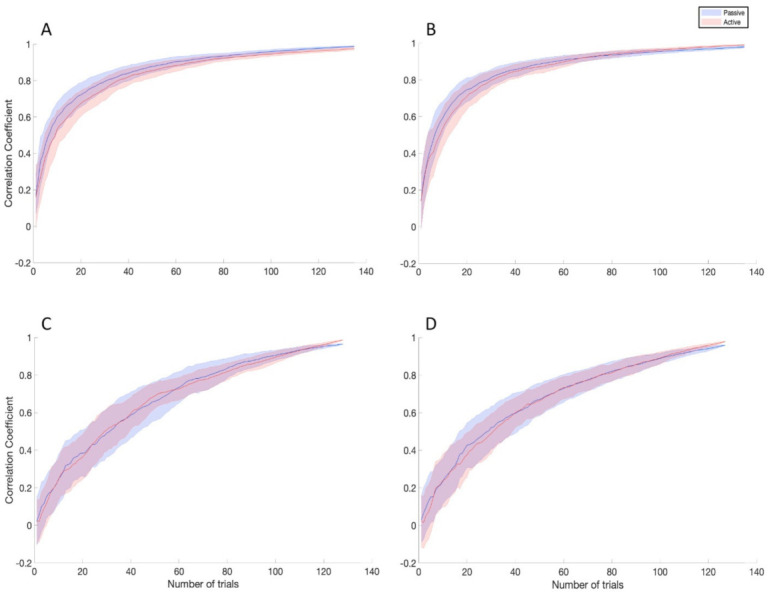
Efficiency comparison between PE (blue lines) and AE (red lines) for M1 ISICA (**A**), M1 SSICA (**B**), mPFC ISICA (**C**), and mPFC SSICA (**D**) conditions. The shaded areas indicate the standard error of the mean. No statistically significant differences were observed in all the conditions tested (see text for details).

For TEP analysis, six time windows were identified, as explained above, for the stimulation of M1 (13–17 ms, 18–32 ms, 33–53 ms, 54–70 ms, 71–120 ms, and 121–350 ms, as illustrated in Figure 3 and Figure 4) and mPFC (13–19 ms, 20–42 ms, 43–59 ms, 60–75 ms, 76–174 ms, and 175–350 ms, as illustrated in Figure 5 and Figure 6). No significant differences were found in the amplitude comparison of TEPs obtained with PE and AE recordings, for both stimulated areas and pre-processing methods (Figure 3, Figure 4, Figure 5 and Figure 6). The CCC analysis yielded very similar results, with only sparse electrodes showing CCC values that were significantly lower than expected, compared with the null hypothesis (Figure 3, Figure 4, Figure 5 and Figure 6). Lastly, no significant differences between sessions were found in the efficiency analysis, for both stimulated areas and pre-processing methods (Figure 7).

## 4. Discussion

Our results did not highlight significant differences in the amplitude and topography of TEPs between PE and AE, for stimulation of the left M1 and right mPFC. Signals collected in the two sessions also showed a high degree of similarity. The different electrode sets performed similarly in terms of approximation of the TEP with increasing numbers of trials. Importantly, these results held true when two different pre-processing methods were used, and they were not biased by differences in location and error in coil positioning.

### 4.1. Comparison between Recording Systems

In this paper, we aimed to directly compare the performance of active and passive EEG recording systems used in conjunction with TMS. No evidence of differences in terms of TEP amplitude and scalp topography were observed between PE and AE, irrespective of the area stimulated and the pre-processing pipeline used. This helped to clarify some potential pitfalls concerning the use of AE in conjunction with TMS, which had possible theoretical support from the work by Laszlo and colleagues [8]. This work suggested that AE offer worse performance when fast voltage changes are involved; we reasoned that this might have represented an issue in the TMS-EEG setting, due to the high frequency content of the cortical signal, especially in the first 70 ms [7]. Apparently, this was not the case, as the signal that was recorded with AE was very similar to that obtained with PE. This result was confirmed by the similarity analysis. CCC values between the two recording conditions were high and, most importantly, were not statistically different when compared to the those obtained under the null hypothesis, apart from few exceptions. We do not think that these influence the validity of our findings, since the electrodes with low CCC were few and, in most cases, distant from the stimulation point. This result should also be considered in the light of the characteristics of the statistical analysis adopted, which was essentially a one-tailed test, designed to increase power for detecting low-correlating electrodes.

To further compare the two recording systems, we computed the similarity between the final TEP, obtained by averaging all the trials collected, and intermediate TEPs obtained by progressively increasing numbers of trials. We did not find statistically significant differences between recordings with PE and AE, meaning that the two systems showed comparable ability of approximating the TEP at any trial number. From an operational point of view, this would mean that experiments planned using similar number of trials would give comparable outcome signals, irrespective of the amplification system used.

### 4.2. Comparison between Pre-Processing Pipelines

In this study, we pre-processed our data in two different ways, using either SSP-SIR or ICA as a first step to remove early, TMS-locked artefacts, mostly consisting of EMG activity due to scalp muscle activation and voltage decay. A consensus on the best pre-processing pipeline has yet to be reached [1]; thus, we deemed it important to collect data about different pre-processing solutions. There are two main reasons why the two pipelines could have yielded different results, as in previous work [24,36]. The first pertains to the EEG artefact that is caused by scalp muscle activation, which is handled differently by SSP-SIR and ICA. We found this artefact to be slightly different between stimulated areas (M1 and mPFC), due to the non-homogeneous arrangement of muscle fibres across the scalp, and also between the two sessions (PE and AE), due to the different TMS intensities and coil to scalp distances. Additionally, PE and AE were previously reported to perform differently when there are sudden voltage changes [8], such as those associated to TMS-induced scalp muscle activity. Despite these potential sources of variability, the two pipelines led to similar results when PE and AE were compared, in all the three analyses used. Additionally, although no formal comparison was made, as it would have been beyond the scope of the present work, no major differences in TEPs topography or amplitude were observed with the two pre-processing methods. Overall, these descriptive results could be considered to be a ‘field test’ for SSP-SIR-based TMS-EEG pre-processing, where this algorithm showed good consistency with the more traditional ICA. Importantly, SSP-SIR does not require the assumption of statistical independence between artefacts and brain signals [25]; this is because, unlike other analysis methods, the source decomposition that SSP performs does not depend on the orthogonality of components or the availability of source or conductivity models [37]. Hence, SSP-SIR may present as a mathematically safer system to the user than ICA, but further studies are necessary to compare the two methods.

## 5. Conclusions and Limitations

The present results allow to conclude that AE can be used in the TMS-EEG setting with outcomes very similar to PE. Importantly, as shown by the efficiency analysis, the two systems require a similar number of trials to approximate the TEP, information that can be useful for experiment planning. We acknowledge that the data were acquired on a limited number of subjects; however, the sample size was slightly larger than that used in a previous, similar investigation [8]. Based on the present outcomes, we suggest that the choice of AE should also be guided by factors other than quality of the recordings. For instance, one advantage of AE is represented by the preamplification process, which decreases electrical line noise. Additionally, AE allow for easier and faster skin preparation, which results in substantially less discomfort for test subjects; this can be particularly valuable for clinical studies, where testing time can be limited by frailty of participants and limited tolerance to experimental procedures. On the other hand, AE are more expensive, and their added thickness entails a higher TMS intensity. This might lead to decreased spatial specificity of stimulation, as well as larger auditory EEG responses, due to the increased volume of the TMS click. In essence, all these factors considered, AE represent a viable solution for TMS-EEG, with advantages and disadvantages, compared to the more classic PE, which should be assessed based on the specific experimental setting.

## Figures and Tables

**Table 1:** Summary of raw values and between-session comparisons related to stimulation intensities, VAS scores, MNI coordinates and coil position error. AE: active electrodes; PE: passive electrodes; VAS: visual analogue scale. MNI coordinates and coil position errors are expressed in mm.

	Motor Cortex				Prefrontal Cortex			
	PE	AE	Z	*p*	PE	AE	Z	*p*
**Stimulation intensity**	41.38 ± 9.21	47.50 ± 10.23	−2.401	0.016	54.60 ± 10.94	62.55 ± 12.06	−2.527	0.012
**VAS score** (**auditory**)	0.38 ± 0.74	1.00 ± 1.07	−1.633	0.102	1.38 ± 0.92	2.13 ± 0.64	−1.890	0.059
**VAS score** (**discomfort**)	0.25 ± 0.46	0.25± 0.71	0	1	0.50 ± 0.93	0.50 ± 0.76	0	1
**MNI coord** (**x**)	−44.09 ± 5.35	−40.60 ± 7.22	−1.260	0.208	14.07 ± 4.69	14.51 ± 6.92	−0.420	0.674
**MNI coord** (**y**)	−10.22 ± 3.48	−13.74 ± 4.47	−1.4	0.161	46.46 ± 6.24	48.45 ± 3.82	−1.120	0.263
**MNI coord** (**z**)	73.21 ± 4.29	76.52 ± 3.11	−1.680	0.093	57.36 ± 6.42	56.35 ± 6.07	0.04	0.97
**Error** (**linear**)	1.73 ± 0.51	1.99 ± 0.29	−0.840	0.401	2.42 ± 1.53	1.90 ± 0.26	−0.70	0.484
**Error** (**angular**)	2.60 ± 1.17	3.14 ± 0.86	−1.260	0.208	3.42 ± 0.84	3.06 ± 1.00	−1.120	0.263
**Error** (**twist**)	3.11 ± 1.94	3.80 ± 1.87	−0.980	0.327	2.19 ± 1.02	2.89 ± 1.86	−0.840	0.401

## Data Availability

Data are available upon request.
